# Sustainable Strategy Using Tung Fruit-Derived Humic Substances–Ferrihydrite for Simultaneous Pollutant Removal and Fertilizer Recovery

**DOI:** 10.3390/toxics13110974

**Published:** 2025-11-12

**Authors:** Hao Lin, Yuhuan Su, Chengfeng Liu, Jiayi Tu, Ruilai Liu, Jiapeng Hu

**Affiliations:** 1 Fujian Provincial Key Laboratory of Eco-Industrial Green Technology, Wuyi University, Wuyishan 354300, China; linhaosg@wuyiu.edu.cn (H.L.); suyuhuan0001@163.com (Y.S.); 2 Fujian Provincial Bamboo Engineering Technology Research Center, Wuyishan 354300, China; 3 College of Resources and Environment, Fujian Agriculture and Forestry University, Fuzhou 350002, China; 15656590901@163.com; 4 College of Environment and Safety Engineering, Fuzhou University, Fuzhou 350001, China; chengfeng5813@163.com

**Keywords:** artificial humic substances, ferrihydrite composites, phosphate removal, adsorption mechanism, resource recovery

## Abstract

Phosphate pollution caused by human activities has become a pressing environmental issue, leading to eutrophication and severe ecological risks. In this study, artificial humic acid (HA) and fulvic acid (FA) were synthesized from tung fruit and glucose, respectively, and further composited with ferrihydrite (Fh) to prepare HA/Fh and FA/Fh adsorbents for phosphate removal. The structural and morphological characteristics of the composites were confirmed by SEM, XRD, FTIR, and XPS analyses, which indicated successful complexation of HA or FA with Fh through ligand exchange and surface interactions. Batch adsorption experiments revealed that HA/Fh and FA/Fh exhibited significantly enhanced adsorption capacities compared to pristine Fh, with maximum Langmuir adsorption capacities of 33.67 mg g^−1^ and 37.06 mg g^−1^, respectively. The adsorption behavior was well described by the pseudo-second-order kinetic model and the Langmuir isotherm, suggesting a chemisorption-dominated process involving ligand exchange between surface –OH groups of Fh and phosphate ions, supplemented by electrostatic attraction. Coexisting ion studies demonstrated that Cl^−^ and SO_4_^2−^ slightly promoted phosphate adsorption, while NO_3_^−^ and CO_3_^2−^ strongly inhibited it, highlighting the competition of multivalent anions with phosphate for Fe^3+^ active sites. Importantly, the phosphate-enriched adsorbents can be directly recycled as phosphorus fertilizers, providing a sustainable pathway for both environmental remediation and phosphorus resource recovery.

## 1. Introduction

Phosphorus is a vital element for biological systems, yet excessive input of phosphorus compounds into aquatic environments, primarily caused by human activities such as agricultural fertilization, domestic sewage discharge, and industrial effluents, has become a major environmental concern [1,2,3]. The continuous enrichment of phosphate in water bodies often leads to eutrophication, algal blooms, oxygen depletion, and subsequent deterioration of water quality, which pose severe risks to aquatic ecosystems and human health. Moreover, long-term exposure to elevated phosphate levels in drinking water has been associated with kidney damage, heart and vascular diseases, and metabolic dysfunctions [4,5]. To mitigate phosphate pollution, a series of treatment strategies have been explored, including chemical precipitation, ion exchange, biological phosphorus removal, membrane separation and adsorption [6,7]. Compared with other methods, chemical precipitation is widely applied but generates large amounts of secondary sludge; biological methods are cost-effective but easily influenced by environmental conditions; and membrane-based processes exhibit high separation efficiency but suffer from high cost and fouling problems. In contrast, adsorption is considered an efficient and sustainable approaches due to its operational simplicity, high removal efficiency, cost-effectiveness, and potential for adsorbent regeneration and reuse [8,9,10].

Ferrihydrite (Fh), a poorly crystalline iron oxyhydroxide, has attracted extensive attention for the efficient adsorption of phosphate [11,12]. Its high surface area, abundant hydroxyl groups, and strong affinity toward phosphate ions enable the establishment of inner-sphere complexes through ligand exchange. Recent studies have demonstrated that ferrihydrite-based materials can significantly enhance phosphate adsorption capacity when combined with organic matter, metal ions, or bio-based modifiers [13,14,15]. Fulvic acid and artificial humic acid fabricated through hydrothermal or oxidative conversion of biomass resources, mimic the structure and functional groups of natural humic substances [16,17]. These artificial humic substances are abundant in oxygen-containing groups such as –COOH, –OH, and C=O, supplying abundant reactive sites for complexation and ligand exchange with phosphate ions. Compared with their natural counterparts, artificial HA and FA can be obtained in a more controllable and sustainable manner, with tunable properties depending on the synthesis conditions. When ferrihydrite is composited with HA or FA, the organic ligands not only improve the dispersion and stability of ferrihydrite particles but also introduce additional functional groups, thereby offering more adsorption sites and synergistically enhancing the overall phosphate uptake capacity. Furthermore, recycling the adsorbent after phosphate capture provides an additional advantage. Phosphate-enriched adsorbents can be directly reused as phosphorus fertilizers, thereby achieving dual benefits of environmental remediation and resource recovery [18,19]. This strategy not only alleviates the problem of phosphorus pollution but also contributes to sustainable phosphorus management in agriculture. It is worth noting that in China, among the three essential nutrients in chemical fertilizers (nitrogen, phosphorus, and potassium), phosphate fertilizers are facing a dual challenge of insufficient phosphorus resources and low utilization efficiency. In particular, the utilization rate of bio-based phosphate fertilizers remains very low, which further exacerbates the pressure on sustainable phosphorus supply. Under this circumstance, the development of efficient adsorbents for phosphate recovery not only addresses environmental pollution but also provides an important pathway for improving fertilizer efficiency and ensuring agricultural sustainability.

In this context, the present work aims to develop novel ferrihydrite composites modified with artificial humic substances derived from tung fruit, and to evaluate their adsorption performance toward phosphate. Special emphasis is placed on understanding the adsorption mechanisms, the influence of environmental factors, and the potential of phosphate-laden materials for direct utilization as fertilizers.

## 2. Materials and Methods

**Figure 1:**
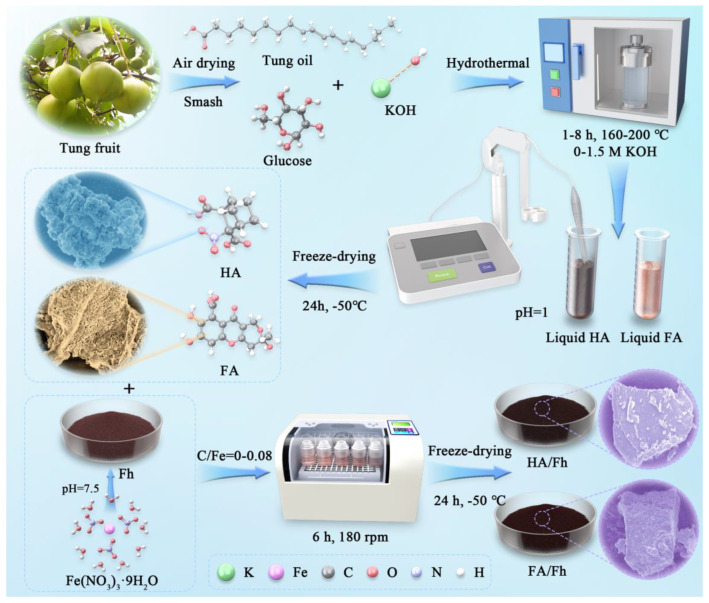
Schematic diagram for the preparation of HA/Fh and FA/Fh adsorbents.

The raw material suppliers and purities are provided in Section S1 of Supplementary Materials. The preparation schemes of artificial humic acid (HA) and artificial fulvic acid (FA), ferrihydrite (Fh), as well as HA/Fh and FA/Fh composites, are shown in Figure 1. For further details, please refer to Section S2 of Supplementary Materials.

## 3. Results and Discussion

### 3.1. Characterization Analysis of HA/Fh and FA/Fh

**Figure 2:**
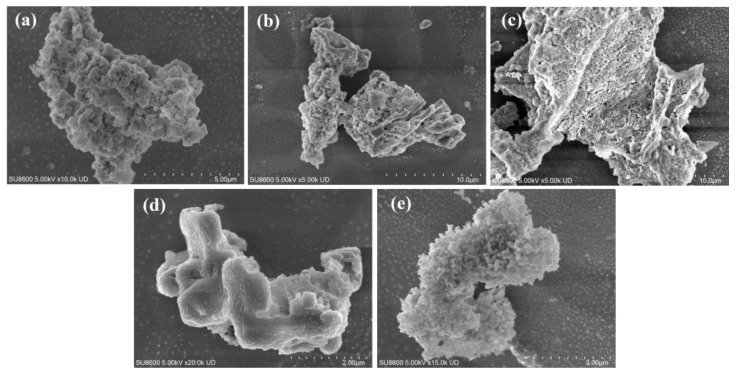
SEM images of (**a**) HA, (**b**) FA, (**c**) Fh, (**d**) HA/Fh adsorbents, (**e**) FA/Fh adsorbents.

Figure 2a shows the SEM image of HA. The surface of HA is rough and exhibits a porous structure with pore sizes ranging from 300 to 500 nm. The formation of these pores can be primarily ascribed to the intrinsic cellular wall structure of tung fruit as a natural biomass material, which inherently contains micron-sized channels and cavities. Under strong alkaline conditions (e.g., KOH), reactions with phenolic hydroxyl, ether, and ester groups in the biomass lead to selective dissolution or chain cleavage of organic matter, leaving behind irregular voids or channels. In addition to the porous morphology, HA also exhibits a certain degree of “stacking.” This phenomenon arises from the presence of abundant aromatic rings (benzene rings, substituted phenyls, and quinone structures) in HA, which possess π-electron cloud overlap capability. During drying or aggregation, these aromatic moieties undergo π-π interactions, driving the ordered alignment and layered stacking of sheets or chains, similar to the stacking behavior observed in graphene or graphite. Figure 2b presents the SEM image of FA, which also displays abundant pores but with denser distribution compared with HA, with pore sizes ranging from 200 to 300 nm. Under alkaline hydrothermal conditions, glucose undergoes thermal decomposition to generate small-molecule intermediates such as carboxylic acids, phenols, and aldehydes/ketones. With the assistance of KOH, these intermediates further undergo condensation, redox reactions, and aromatization to form low-molecular-weight conjugated polymers enriched with aromatic structures and functional groups (e.g., –COOH, –OH, and C=O). Subsequent pH adjustment to acidic conditions enables FA precipitation, yielding a product with abundant polar groups, good water solubility, and structural stability [20]. The porous structure of FA is primarily formed during the release of gases (e.g., CO_2_, CO, and H_2_O vapor) generated in the decomposition process, which escape through the polymer matrix and create internal and surface pores. Meanwhile, condensation, aromatization, and cross-linking reactions under alkaline catalysis give rise to an irregular three-dimensional molecular framework with inherent porosity. Figure 2c shows ferrihydrite synthesized by the hydrolysis method, exhibiting loose blocky porous morphology, indicating its amorphous or poorly crystalline nature as an iron oxide precipitate. Figure 2d,e present the SEM images of HA/Fh and FA/Fh adsorbents, respectively. After compositing, both HA/Fh and FA/Fh maintained porous structures and displayed partial stacking. Such porous morphologies are beneficial for increasing the BET surface area, which offers more active adsorption sites, thereby improving the phosphate adsorption capacity of the materials [21].

**Figure 3:**
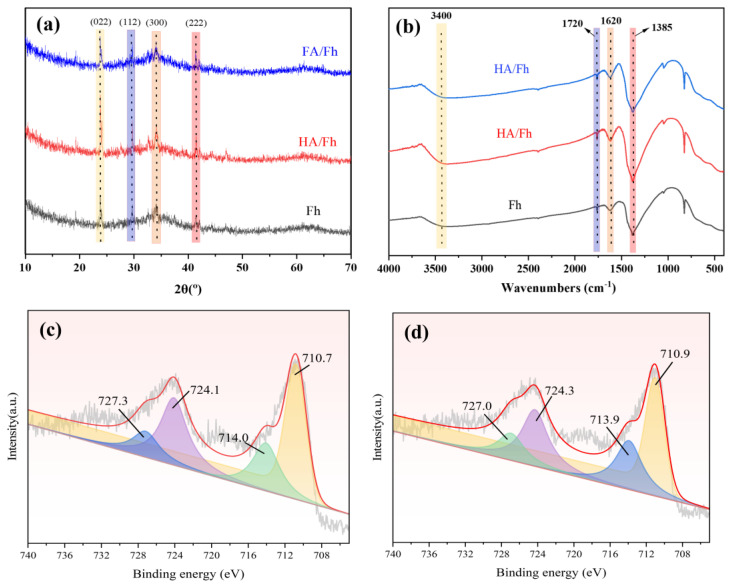
(**a**) XRD pattern, (**b**) FTIR spectra of Fh, HA/Fh and FA/Fh; XPS spectrum of Fe2p, (**c**) Fh, (**d**) FA/Fh.

Figure 3a shows the XRD patterns of HA/Fh and FA/Fh adsorbents. Fh exhibits weak and broad diffraction peaks at 2θ = 23.79°, 29.75°, 34.14°, and 41.50°, which correspond well with the JCPDS standard card (PDF#29-0715) and can be assigned to the (002), (112), (300), and (222) planes, characteristic of six-line ferrihydrite [22]. The other two diffraction peaks were not observed, mainly due to the low crystallinity of the six-line ferrihydrite. This indicates that the ferrihydrite synthesized by the hydrolysis method in this study is a six-line ferrihydrite with a highly dispersed or partially amorphous nanostructure, which facilitates coordination with organic ligands such as HA and FA and is therefore commonly used in adsorptive materials. After compositing with HA, the peak positions of ferrihydrite remained unchanged, but the peak intensities became weaker. This reduction in intensity may be attributed to the coordination between Fe^3+^ ions and the active groups (–COOH and –OH) in HA, forming HA–Fe complexes and thus weakening the diffraction signals of ferrihydrite. Figure S1 shows the FTIR spectra of HA and FA. All samples exhibit a broad and strong absorption band around 3400 cm^−1^, corresponding to the O–H stretching vibration, indicating the presence of abundant hydroxyl groups in HA and FA. The absorption peaks observed at approximately 2920–2850 cm^−1^ are assigned to the C–H stretching vibrations of –CH_3_ and –CH_2_, suggesting the existence of alkyl chains in humic substances. A strong absorption peak at 1720 cm^−1^ is attributed to the C=O stretching vibrations of carboxylic acids, esters, and carbonyl groups, which are the main sources of acidic functional groups in HA and FA. A broad band appearing in the range of 1250–1030 cm^−1^ is associated with the stretching vibrations of C–O, C–N, and C–C bonds. Figure 3b presents the FTIR spectra of HA/Fh and FA/Fh adsorbents. No significant changes in the positions of the major absorption bands were observed after compositing with Fh. In the spectrum of HA/Fh, the bands at 3400 cm^−1^ and 1620 cm^−1^ correspond to the stretching vibration of –OH groups, while the peak at 1720 cm^−1^ is assigned to the C=O stretching vibration of carboxylic acids, esters, or carbonyl groups. In addition, a strong band at 1385 cm^−1^ is observed, which can be ascribed to NO_3_^−^ residues originating from incomplete washing during the preparation of ferrihydrite [23]. The symmetric stretching vibration peak of –COO^−^ groups in HA and FA at 1406 cm^−1^ disappeared after compositing, which is mainly due to ligand exchange between surface –OH groups of Fh and –COO^−^ groups of HA or FA [24]. This ligand exchange represents a strong binding mode, which not only enhances the stability of the composite adsorbents but also prevents the easy separation of HA and FA from Fh. Figure 3c,d show the Fe 2p XPS spectra. The Fe 2p signals exhibit peaks at binding energies of 710.7 eV, 714.0 eV, 724.1 eV, and 727.3 eV. The peaks at 710.7 eV and 724.1 eV are assigned to the Fe^3+^ 2p_3_/_2_ and 2p_1_/_2_ main peaks, respectively, while those at 714.0 eV and 727.3 eV correspond to the satellite peaks of Fe^3+^, indicating that Fe exists in a high-spin state with a large number of unpaired electrons. After compositing with FA, these characteristic Fe 2p peaks remained, confirming that Fe in Fh still exists in the Fe^3+^ state. However, noticeable shifts in peak positions were observed, suggesting the formation of chemical bonds between HA (or FA) and Fh, possibly involving Fe–O–C or Fe–O–COOH coordination structures.

### 3.2. Adsorption Experiments

**Figure 4:**
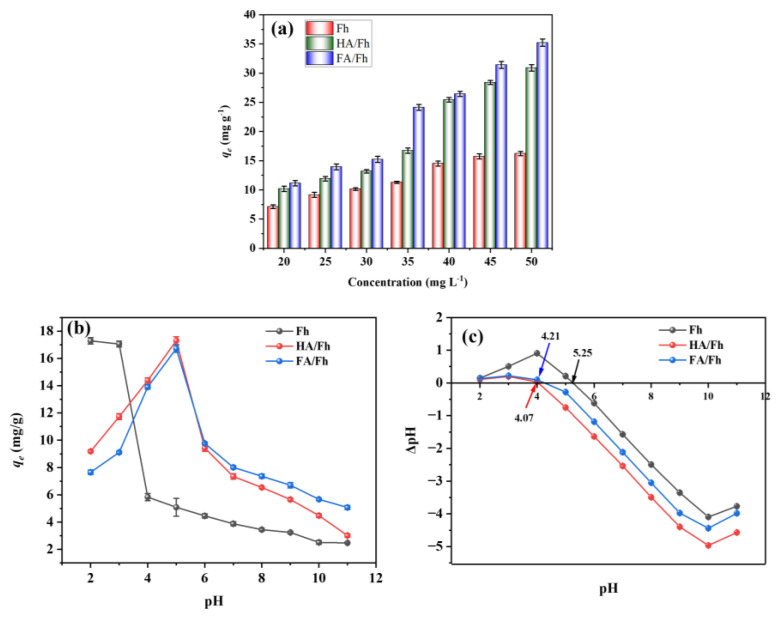
(**a**) Adsorption capacity of phosphate by HA/Fh and FA/Fh adsorbents, (**b**) the effect of pH on the adsorption capacity, (**c**) the point of zero charge (pH_zpc_) of the adsorbents.

Different initial phosphate concentrations were adsorbed using Fh, HA/Fh, and FA/Fh, and the detailed experimental procedures are provided in Section S3 of the Supplementary Materials. Figure 4a illustrates the adsorption capacities of Fh, HA/Fh, and FA/Fh for phosphate. At an initial phosphate concentration of 20 mg L^−1^, the adsorption capacities of Fh, HA/Fh, and FA/Fh were 7.13 ± 0.34, 10.19 ± 0.45, and 11.14 ± 0.45 mg g^−1^, respectively. With increasing phosphate concentration, the adsorption capacity of all three materials gradually increased. This can be explained by the intrinsic nature of the adsorption process, which involves the interaction between solute ions and active sites on the adsorbent surface. At higher phosphate concentrations, more phosphate ions are available in the solution, leading to an increased probability of contact and collision with adsorbent sites, thereby enhancing mass transfer driving force and concentration gradient and promoting the fixation of more ions on the adsorbent surface [25]. When the concentration reached 50 mg L^−1^, the adsorption capacities of Fh, HA/Fh, and FA/Fh increased to 16.23 ± 0.38, 30.91 ± 0.56, and 35.23 ± 0.62 mg g^−1^, respectively. At all concentrations tested, HA/Fh and FA/Fh exhibited significantly higher adsorption capacities than Fh, which can be attributed to the synergistic effect between HA or FA and Fh. The organic coatings of HA or FA improved the dispersion and surface area of Fh, while introducing additional functional groups that provided extra binding sites for phosphate ions [26,27]. Figure 4b shows the effect of pH on phosphate adsorption. For Fh, the adsorption capacity was 17.33 ± 0.23 mg g^−1^ at pH 2 and decreased with increasing pH. In contrast, HA/Fh and FA/Fh showed lower adsorption capacities of 9.20 ± 0.10 mg g^−1^ and 7.65 ± 0.16 mg g^−1^ at pH 2, but their capacities increased with pH and reached maximum values of 17.23 ± 0.29 mg g^−1^ and 16.77 ± 0.26 mg g^−1^ at pH 5. This behavior is closely related to the point of zero charge (pH_zpc_) of the adsorbents, which were determined to be 4.07 and 4.21 for HA/Fh and FA/Fh, respectively (Figure 4c). When pH < pH_zpc_, the adsorbents surface is positively charged, favoring the adsorption of negatively charged phosphate ions. Conversely, at pH > pH_zpc_, the increasing concentration of OH^−^ in solution competes with phosphate ions for adsorption sites on the adsorbent surface, leading to a decline in adsorption capacity [25].

### 3.3. Adsorption Kinetics

**Figure 5:**
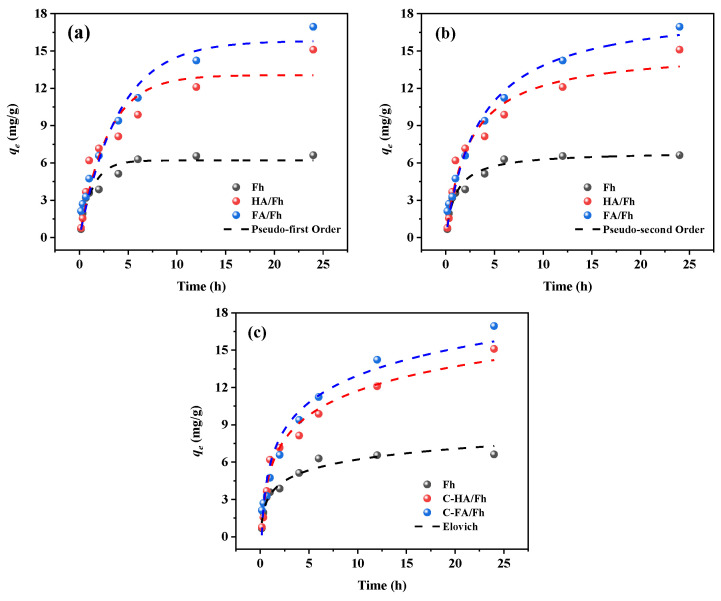
Adsorption kinetics by HA/Fh and FA/Fh adsorbents: (**a**) pseudo-first-order kinetic models, (**b**) pseudo-second-order kinetic models, (**c**) Elovich models.

The adsorption efficiency of phosphate ions by Fh, HA/Fh, and FA/Fh was measured at different time intervals. The adsorption process was fitted using the pseudo-first-order, pseudo-second-order, and Elovich models. The detailed experimental procedures, fitting equations, and corresponding parameters are provided in Section S4 of the Supplementary Materials. Adsorption kinetics describe the variation in adsorption capacity with time, providing insights into the adsorption rate and kinetic behavior. By introducing kinetic models, the equilibrium time, rate constants, and controlling mechanisms of the adsorption process can be determined. Furthermore, adsorption kinetics allow inference of the adsorption mechanism, including surface molecular diffusion and the saturation of adsorption sites. In this study, the pseudo-first-order, pseudo-second-order, and Elovich models were employed to fit the kinetic data, and the results are presented in Figure 5, with the corresponding parameters summarized in Table 1. The correlation coefficients (*R*^2^) of all three models were greater than 0.9, indicating that each model could adequately describe the adsorption system. Comparatively, the *R*^2^ values of the pseudo-second-order model for Fh, HA/Fh, and FA/Fh were all higher than 0.95, suggesting that this model provided the best fit among the three. The equilibrium adsorption capacities (*qₑ*) of Fh, HA/Fh, and FA/Fh calculated by the pseudo-second-order model were 6.91, 17.67, and 16.97 mg g^−1^, respectively, which were in close agreement with the experimental values (*q_exp_*) of 6.21, 17.23, and 16.77 mg g^−1^. This consistency further verified the high applicability of the pseudo-second-order model to the adsorption process. In this model, the adsorption rate is considered proportional to the square of the available adsorption sites, suggesting that it is influenced not only by the site quantity but also by the interactions among them, thus capturing the complexity of the process. Consequently, the adsorption of phosphate onto Fh, HA/Fh, and FA/Fh was predominantly governed by chemisorption kinetics, accompanied by electrostatic interactions between the adsorbent and phosphate ions. This suggests that ion exchange contributed significantly to, in which phosphate ions replaced –OH groups on the surface of Fh to form inner-sphere complexes [28,29].

### 3.4. Adsorption Isotherms

**Figure 6:**
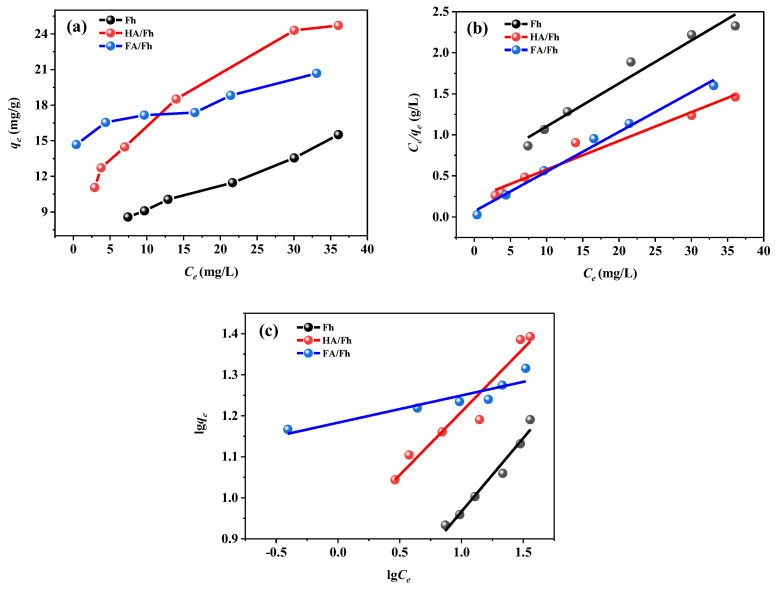
Adsorption thermodynamics of HA/Fh and FA/Fh of adsorbents: (**a**) adsorption isothermal curve, (**b**) Langmuir model, (**c**) Freundlich model.

The adsorption isotherm experiments of phosphate ions by Fh, HA/Fh, and FA/Fh were conducted under different initial concentrations. The detailed experimental procedures are provided in Section S5 of the Supplementary Materials. The adsorption processes were fitted using the Langmuir and Freundlich isotherm models, and the meanings of all fitting parameters are also described in Section S5 of the Supplementary Materials. Adsorption temperature is one of the key factors influencing adsorption behavior, as it affects adsorption thermodynamics, kinetics, equilibrium, and the interactions between adsorbents and adsorbates. Phosphate adsorption on Fh, HA/Fh, and FA/Fh was examined, and the resulting data were analyzed using the Langmuir and Freundlich models. The fitting results are shown in Figure 6, and the calculated parameters are summarized in Table 2. In the Langmuir model, adsorption is assumed to occur as a monolayer process on a homogeneous surface, where adsorbed ions form a uniform layer on the adsorbent surface. In contrast, the Freundlich model assumes that adsorption is not restricted to a monolayer, and that adsorption capacity decreases with increasing surface coverage; thus, it is more suitable for multilayer adsorption on heterogeneous surfaces. The fitting results demonstrated that the correlation coefficients (*R*^2^) of the Langmuir model were all higher than 0.96, and significantly greater than those of the Freundlich model, suggesting that phosphate adsorption onto Fh, HA/Fh, and FA/Fh followed a monolayer adsorption mechanism, with the adsorbent surfaces forming uniformly distributed adsorption layers. Based on the Langmuir model, the theoretical maximum adsorption capacities of Fh, HA/Fh, and FA/Fh for phosphate were calculated to be 17.63, 33.67, and 37.06 mg g^−1^, respectively. The Freundlich model is an empirical equation in which the parameter 1/n reflects the adsorption intensity and the heterogeneity of the adsorbent surface. When 1/n < 1, it indicates favorable adsorption performance [30]. For Fh, HA/Fh, and FA/Fh, the values of n were all greater than 1, corresponding to 1/n < 1, thereby further confirming that these materials exhibit excellent adsorption performance toward phosphate.

### 3.5. Effect of Coexisting Ions

**Figure 7:**
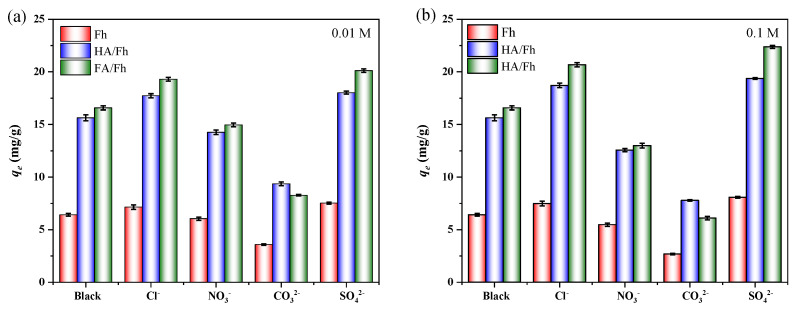
Effect of coexisting ions on phosphate adsorption performance: (**a**) 0.01 M, (**b**) 0.1 M.

In real wastewater treatment scenarios, the water matrix is highly complex, and coexisting ions or organic compounds can markedly affect the adsorption performance of the adsorbents. Therefore, it is necessary to investigate the effect of coexisting ions on phosphate removal. The experimental procedures for studying the effects of coexisting ions such as Cl^−^, NO_3_^−^, CO_3_^2−^, and SO_4_^2−^ on the adsorption of phosphate ions are provided in Section S6 of the Supplementary Materials. Figure 7 presents the influence of common ions such as Cl^−^, NO_3_^−^, CO_3_^2−^, and SO_4_^2−^ on the adsorption of phosphate. The results showed that Cl^−^ and SO_4_^2−^ promoted phosphate adsorption, whereas NO_3_^−^ and CO_3_^2−^ exhibited clear inhibitory effects. For example, when using FA/Fh as the adsorbents, the adsorption capacity for phosphate decreased by 63.24% in the presence of 0.1 M CO_3_^2−^. This can be attributed to the fact that both CO_3_^2−^ and PO_4_^3−^ are multivalent anions with high charge density and share similar adsorption mechanisms, primarily involving electrostatic attraction and the formation of inner-sphere complexes with Fe^3+^ active sites. At higher concentrations, CO_3_^2−^ preferentially occupies the positively charged sites on the adsorbent surface, thereby competing with PO_4_^3−^ for adsorption and severely suppressing phosphate uptake.

### 3.6. Adsorption Mechanism

**Figure 8:**
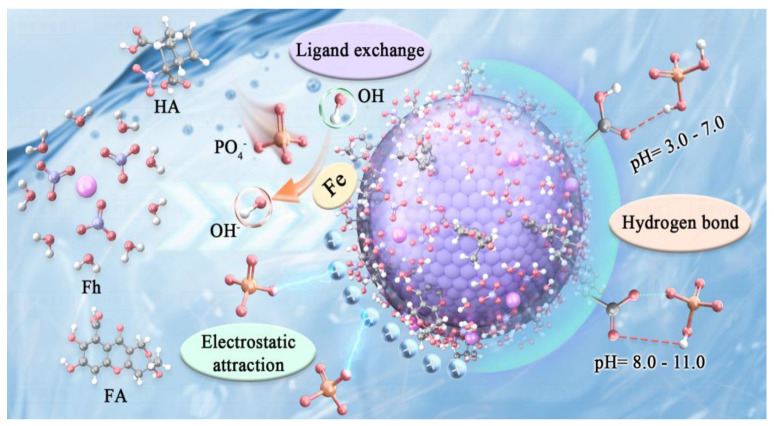
Adsorption mechanism of phosphate by HA/Fh and FA/Fh composite.

Derived from the results of investigation of adsorption rates and isotherm behavior, the adsorption mechanism of phosphate onto HA/Fh and FA/Fh adsorbents can be further elucidated. The experimental data demonstrated that these composites not only exhibited high adsorption capacities and rapid adsorption rates but also fit well with the pseudo-second-order kinetic model and the Langmuir monolayer isotherm model. These findings indicate that the adsorption process is mainly governed by chemisorption. Specifically, the surfaces of HA/Fh and FA/Fh are rich in hydroxyl (–OH) functional groups, which can undergo ligand exchange with phosphate ions in solution. In this process, the oxygen atoms of phosphate replace surface –OH groups and coordinate with Fe atoms, forming stable inner-sphere complexes. Meanwhile, electrostatic interactions between the charged surface of the adsorbents and the negatively charged phosphate ions further promote the enrichment and fixation of phosphate onto the adsorbent surface. These two mechanisms act synergistically, resulting in enhanced adsorption efficiency and selectivity of the adsorbents. As illustrated in Figure 8, phosphate ions are chemically bound to the adsorbent surface via hydroxyl substitution, while electrostatic attraction accelerates their accumulation, leading to the superior adsorption performance of HA/Fh and FA/Fh compared with pristine Fh. This synergistic mechanism not only explains the excellent phosphate removal performance of the composites but also provides a theoretical basis for further structural optimization and improvement of their environmental remediation efficiency.

## 4. Conclusions

In this study, artificial humic acid (HA) and fulvic acid (FA) were successfully synthesized from tung fruit and glucose, respectively, and further composited with ferrihydrite (Fh) to prepare HA/Fh and FA/Fh adsorbents. Structural characterizations confirmed the successful incorporation of organic functional groups into ferrihydrite, which improved its dispersion, surface area, and stability. Batch adsorption experiments demonstrated that HA/Fh and FA/Fh exhibited significantly higher phosphate adsorption capacities than pristine Fh, with maximum Langmuir adsorption capacities of 33.67 mg g^−1^ and 37.06 mg g^−1^, respectively. Kinetic analyses indicated that the adsorption process was best fitted by the pseudo-second-order model, while isotherm studies revealed that the adsorption followed the Langmuir model, suggesting a monolayer chemisorption process. The adsorption mechanism was mainly attributed to ligand exchange between the –OH groups on ferrihydrite and phosphate ions, supplemented by electrostatic attraction. Coexisting ion experiments showed that Cl^−^ and SO_4_^2−^ promoted phosphate adsorption, whereas NO_3_^−^ and CO_3_^2−^ suppressed it, with CO_3_^2−^ showing the strongest competitive effect. Overall, this work highlights the superior performance of HA/Fh and FA/Fh composites for phosphate removal from aqueous solutions. Moreover, the phosphate-laden adsorbents can be directly recycled as phosphorus fertilizers, offering a sustainable strategy for simultaneous water remediation and phosphorus resource recovery.

## Figures and Tables

**Table 1:** Kinetic model parameters for phosphate adsorption by HA/Fh and FA/Fh adsorbents.

Models	Parameter	Fh	HA/Fh	FA/Fh
Pseudo-first Order	*R* ^2^	0.9219	0.9065	0.9553
	*q_e_* (mg g^−1^)	6.203	13.063	15.818
	*K*_1_	0.758	0.344	0.249
Pseudo-second Order	*R* ^2^	0.9663	0.9552	0.9806
	*q_e_* (mg g^−1^)	6.914	17.671	16.967
	*K*_2_	0.141	0.028	0.015
Elovich	*R* ^2^	0.9585	0.9789	0.9523
	A	0.804	0.353	0.319
	β	18.3310	17.864	19.603

**Table 2:** Adsorption isotherm parameters for phosphate adsorption by HA/Fh and FA/Fh adsorbents.

Sample	Langmuir Equation			Freundlich Equation		
	*q_max_* (mg g^−1^)	*K_L_* (L mg^−1^)	*R* ^2^	*K_f_* (L g^−1^)	n	*R* ^2^
Fh	17.634	0.057	0.9934	3.011	0.379	0.9832
HA/Fh	33.669	0.023	0.9968	2.192	0.586	0.9917
FA/Fh	37.056	0.032	0.9962	3.121	0.513	0.9866

## Data Availability

The original contributions presented in this study are included in the article/Supplementary Materials. Further inquiries can be directed to the corresponding author.

## Supplementary Materials

The following supporting information can be downloaded at: https://www.mdpi.com/article/10.3390/toxics13110974/s1, Section S1. Materials. Section S2. Preparation method. Section S3. Adsorption Experiments of Phosphate. Section S4. Adsorption kinetics. Section S5. Adsorption isotherms. Section S6. Coexisting ion experiments. Section S7. Determination of the point of zero charge (pHzpc). Section S8. Characterization. Figure S1 FTIR spectra of HA and FA. (References [31,32] are cited in the Supplementary Materials)
