# Supplementary material for: Sustainable Strategy Using Tung Fruit-Derived Humic Substances–Ferrihydrite for Simultaneous Pollutant Removal and Fertilizer Recovery
Source: Toxics. 2025 Nov 12;13(11):974. doi: 10.3390/toxics13110974 (PMC12656244; doi:10.3390/toxics13110974)
Supplement: Supplementary file 1 [file toxics-13-00974-s001.zip › toxics-3946755-supplementary.pdf]

Supplementary Materials

Sustainable Strategy Using Tung Fruit-Derived Humic Substances–Ferrihydrite for Simultaneous Pollutant Removal and Fertilizer Recovery

Hao Lin [1,2,3], Yuhuan Su [1,2], Chengfeng Liu [4], Jiayi Tu [3], Ruilai Liu [1,2,*] and Jiapeng Hu [1,2,4,*]

[1] Fujian Provincial Key Laboratory of Eco-Industrial Green Technology, Wuyi University, Wuyishan 354300, China
[2] Fujian Provincial Bamboo Engineering Technology Research Center, Wuyishan 354300, China
[3] College of Resources and Environment, Fujian Agriculture and Forestry University, Fuzhou 350002, China
[4] College of Environment and Safety Engineering, Fuzhou University, Fuzhou 350001, China

S1. Materials

Hydrochloric acid (HCl, AR), sodium hydroxide (NaOH, AR), potassium hydroxide (KOH, AR), ferric nitrate nonahydrate ($Fe(NO_3)_3 \cdot 9H_2O$, AR), potassium dihydrogen phosphate (KH_2PO_4, AR), ammonium molybdate, ascorbic acid ($C_6H_8O_6$, AR), sulfuric acid (H_2SO_4, AR), sodium fluoride (NaF, AR), glacial acetic acid (CH_3COOH, AR), sodium chloride (NaCl, AR), sodium carbonate (Na_2CO_3, AR), sodium nitrate ($NaNO_3$, AR), and sodium sulfate (Na_2SO_4, AR) were all of analytical reagent grade and purchased from Sinopharm Chemical Reagent Co., Ltd. (Shanghai, China).

S2. Preparation method

S2.1. Preparation of artificial humic acid (HA)

Artificial humic acid was synthesized from tung fruit, a by-product of oil processing (collected from a tung oil factory in Xiangxi, Hunan, China). The raw material was air-dried and ground to a particle size ≤ 2 mm. Pre-treatment was carried out as follows: the powder was washed with deionized water (solid-to-liquid ratio 1:10, stirred for 30 min) to remove surface oil and soluble impurities, and subsequently dried in an oven at 60 °C to a constant weight before sealed storage. The hydrothermal humification reaction was performed in a 100 mL Teflon-lined stainless-steel autoclave. Specifically, 5.0 g of pretreated tung fruit powder was dispersed in 50 mL of deionized water to form a suspension, followed by the addition of NaOH solution (1 mol L^{-1}). The sealed reactor was heated at 160 °C for 4 h. After cooling to room temperature, the reaction mixture was centrifuged to achieve solid–liquid separation. The pH of the supernatant was adjusted to 1 with HCl to precipitate humic acid. The precipitate was collected by centrifugation and filtration and subsequently freeze-dried at −50 °C under vacuum for 24 h to obtain the final product, denoted as HA.

S2.2. Preparation of artificial fulvic acid (FA)

Artificial fulvic acid was prepared via a hydrothermal route using glucose as the carbon source. Briefly, glucose was dissolved in deionized water at a mass ratio of 1:10 under magnetic stirring at 60 °C. NaOH solution was then slowly added to reach a concentration of 1 mol L^{-1}, yielding a dark-brown transparent solution. The mixture was transferred into a 100 mL Teflon-lined autoclave and subjected to hydrothermal treatment at 200 °C for 2 h. After natural cooling, the brown solution obtained was centrifuged at 8000 rpm for 10 min to remove unreacted carbonized particles. The supernatant was filtered through a 0.22 μm microporous membrane. The pH was adjusted to 1 with HCl to precipitate fulvic acid, which was collected by centrifugation and filtration. The product was freeze-dried at −50 °C under vacuum for 24 h to yield a light-yellow powder, denoted as FA.

S2.3. Preparation of ferrihydrite (Fh)

Ferrihydrite was synthesized by a precipitation method. In a typical procedure, 20 g of $Fe(NO_3)_3·9H_2O$ was dissolved in 300 mL of deionized water under mechanical stirring. 1.0 mol L^{-1} NaOH solution was then slowly added dropwise until the pH reached 7.5. During this process, Fe^{3+} ions were hydrolyzed to form ferric hydroxide complexes, resulting in an orange-red gelatinous precipitate. The suspension was stirred at 150 rpm for 45 min at room temperature, followed by standing until solid–liquid separation was apparent. The precipitate was washed repeatedly with deionized water until the conductivity of the filtrate was <20 μS/cm, then centrifuged and freeze-dried at −50 °C. The obtained sample was stored in a dark and dry environment at 4 °C and denoted as Fh.

S2.4. Preparation of HA/Fh and FA/Fh adsorbents

HA/Fh adsorbents were synthesized by a coprecipitation method. Typically, 20 g of $Fe(NO_3)_3·9H_2O$ was dissolved in 250 mL of deionized water, into which 50 mL of HA solution (containing 0.1776 g HA) was added dropwise under constant stirring. Simultaneously, 1 mol L^{-1} KOH solution was slowly added to adjust the pH to 7.5. The mixture was stirred under dark conditions for 6 h, followed by centrifugation to separate the solid product. The solid was washed repeatedly with deionized water until the conductivity was below 20 μS cm^{-1}. The purified product was freeze-dried to obtain HA/Fh. FA/Fh composites were prepared following the same procedure, with FA replacing HA.

S3. Adsorption Experiments of Phosphate

A phosphate stock solution of 500 mg L^{-1} (prepared from KH_2PO_4) was first prepared and subsequently diluted with deionized water to obtain working solutions with concentrations ranging from 20 to 50 mg L^{-1}. The adsorption experiments were conducted in 50 mL Erlenmeyer flasks, with 20 mL of phosphate solution (20, 25, 30, 35, 40, 45, and 50 mg L^{-1}) and 0.02 g of Fh, HA/Fh, or FA/Fh adsorbent added into each centrifuge tube. The flasks were placed

in a thermostatic shaker and agitated at 150 rpm for 24 h at 25 °C to ensure sufficient adsorption. After equilibrium, the supernatant was immediately withdrawn and filtered through a 0.45 μm membrane. The concentration of phosphate ions was quantified by combining a specified volume of the filtrate with 25 mL of distilled water, 3 mL of ascorbic acid, and 2 mL of ammonium molybdate in a 50 mL volumetric flask, which was subsequently filled to the mark with distilled water. The mixture was then incubated in a water bath at 30 °C for 30 min. The phosphate ion concentration was determined using the molybdenum blue method at 710 nm with a UV-Vis spectrophotometer. The adsorption capacity of the adsorbents was calculated according to Eq. (1).

$$q_e = \frac{v(C_0 - C_e)}{m} \quad \text{(S1)}$$

where q_e (mg g^{-1}): the equilibrium adsorption capacity; V (L): the volume of the initial solution; C_0 and C_e (mg L^{-1}): the initial and equilibrium concentrations, respectively; m (g): the mass of the adsorbent dosage.

To investigate the effect of pH on adsorption efficiency, the pH of the adsorption solutions was adjusted using 0.1 M HCl and NaOH solutions, and the adsorption capacity was determined following the same procedure described above.

S4. Adsorption kinetics

For the kinetic experiments, 0.02 g of adsorbent was added into 20 mL of phosphate solution with an initial concentration of 30 mg/L at pH 5.0. The experiments were performed at 25 °C. At predetermined time intervals, samples were withdrawn, immediately filtered through a 0.45 μm membrane, and analyzed. The residual phosphate concentration was determined using a UV–Vis spectrophotometer at 700 nm to evaluate the adsorption kinetics of phosphate onto the adsorbents.

Adsorption kinetics were analyzed by fitting the experimental data with pseudo-first-order, pseudo-second-order, and intraparticle diffusion models to elucidate the adsorption mechanism. Adsorption kinetics mainly describe the dynamic behavior of adsorbates on the adsorbent surface during the adsorption process, including adsorption rate, influencing factors, and the underlying mechanisms. In this study, the kinetic data were fitted using the pseudo-first-order, pseudo-second-order, and intraparticle diffusion models. The corresponding equations are expressed as follows [1, 2]：

Pseudo-first-order model:

$$\ln(q_e - q_t) = \ln q_e - k_1 t \quad \text{(S2)}$$

Pseudo-second-order model:

$$\frac{t}{q_t} = \frac{1}{k_2 q_e^2} + \frac{t}{q_e} \quad \text{(S3)}$$

Elovich model:

$$q_t = \frac{1}{\beta}\ln(\alpha\beta) - \frac{1}{\beta}\ln(t) \qquad \text{(S4)}$$

where q_t (mg g^{-1}) is the adsorption capacity at time t (min), q_e (mg g^{-1}) is the equilibrium adsorption capacity, k_1 (min^{-1}) is the pseudo-first-order rate constant, k_2 (g mg^{-1} min^{-1}) is the pseudo-second-order rate constant, α ($g \cdot mg^{-1} \cdot min^{-2}$) and β ($mg \cdot g^{-1} \cdot min^{-1}$) are the parameters of the Elovich model, and t (min) represents the adsorption time.

S5. Adsorption isotherms

To investigate the adsorption performance of different materials, a phosphate stock solution of 500 mg/L was first prepared and subsequently diluted with deionized water to obtain solutions with concentrations of 15, 20, 25, 30, 40, and 50 mg/L. In each experiment, 0.02 g of adsorbent was mixed with 20 mL of phosphate solution at pH 5.0, and the temperature was maintained at 25 °C. The adsorption experiments were conducted in 50 mL centrifuge tubes, which were placed in a thermostatic shaker and agitated at 150 rpm for 24 h to ensure sufficient adsorption. After equilibration, the supernatants were immediately collected and filtered through a 0.45 μm membrane. The phosphate concentrations in the filtrates were then measured by UV–Vis spectrophotometry at 700 nm to evaluate the adsorption isotherm characteristics.

The experimental data were fitted using the Langmuir and Freundlich isotherm models, expressed as follows [1, 2]:

Langmuir model:

$$\frac{C_e}{q_e} = \frac{1}{K_L q_m} + \frac{C_e}{q_m} \qquad \text{(S5)}$$

Freundlich model:

$$\lg q_e = \lg K_f + \frac{1}{n}\lg C_e \qquad \text{(S6)}$$

where q_e (mg g^{-1}) is the equilibrium adsorption capacity, q_m (mg g^{-1}) is the maximum adsorption capacity, C_e (mg L^{-1}) is the equilibrium concentration, b (L mg^{-1}) is the Langmuir constant, K_f (mg g^{-1}) is the Freundlich adsorption constant, and n represents adsorption intensity.

S6. Coexisting ion experiments

To assess the tolerance of the adsorbents toward coexisting ions, a 500 mg/L phosphate stock solution was first prepared and diluted to 30 mg/L. Subsequently, different interfering ions, including Cl^-, NO_3^-, SO_4^{2-}, and CO_3^{2-}, were introduced into the solutions at concentrations of 0.01 M and 0.1 M to simulate complex ionic environments in natural waters. Each experiment was carried out in a 50 mL Erlenmeyer flask containing 20 mL of 30 mg/L phosphate

solution and 0.02 g of adsorbent. The flasks were placed in a thermostatic shaker at 25 °C and agitated at 150 rpm for 24 h. After equilibrium, the supernatants were withdrawn and filtered immediately through a 0.45 μm membrane. The residual phosphate concentrations were determined at 700 nm using a UV–Vis spectrophotometer to evaluate the adsorption performance of different materials under ionic interference.

S7. Determination of the point of zero charge (pH_{zpc})

The point of zero charge (pH_{zpc}) of the adsorbents was determined using the pH drift method. In brief, 50 mL of 0.01 mol·L^{-1} NaCl solution was placed in a series of conical flasks, and the initial pH (pH_0) was adjusted to values between 2 and 12 using 0.1 mol·L^{-1} HCl or NaOH. Then, 0.05 g of the adsorbent was added to each flask, and the suspensions were shaken at 25 °C for 24 h to reach equilibrium. The final pH (pH_f) of each solution was recorded. The difference ($\Delta pH = pH_f - pH_0$) was plotted against the initial pH. The pH value at which $\Delta pH = 0$ was taken as the pH_{zpc} of the adsorbent.

S8. Characterization

The morphology and composition of the adsorbent were determined by scanning electron microscopy (SEM, Hitachi SU8010, Japan). The powder X-ray diffraction (XRD) pattern was recorded using Bruker D8 ADVANCE with CuK_α to examined crystal structure of the adsorbent, where the specific angle was selected from 5−70° and the scanning speed was selected at 10 °/min; The surface property of adsorbent was examined using FTIR (Perkin Elmer) in the range 400−4000 cm^{-1} using the KBr disc method; The elemental compositions and chemical valences were determined by XPS (ESCALAB250, Thermo VG, USA).

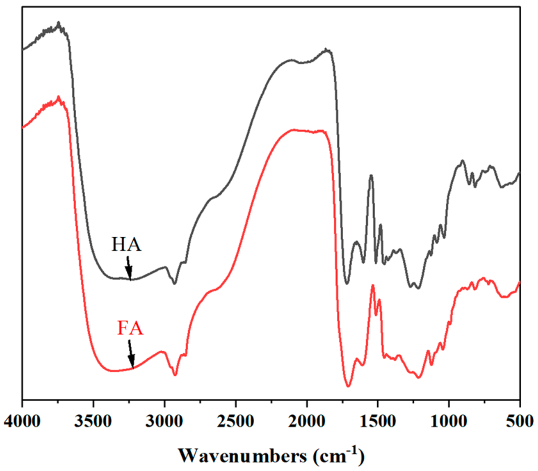

Figure S1 FTIR spectra of HA and FA

References

1. Song, J.; Yu, Y.; Han, X.; Yang, W.; Pan, W.; Jian, S.; Duan, G.; Jiang, S.; Hu, J., Novel MOF (Zr)-on-MOF (Ce) adsorbent for elimination of excess fluoride from aqueous solution. *J. Hazard. Mater.* **2024,** *463*, 132843.
2. Liu, R.; Song, J.; Zhang, Z.; Ji, L.; Yang, W.; Zhao, J.; Jian, S.; Hu, J.; Ma, J., Needle-like PVP@ Ce/Zr-MOFs for the highly efficient selective of fluoride and phosphate from aqueous solution. *Separation Purification Technology* **2025**, 133267.
